# Using MRI to plan breast-conserving surgery following neoadjuvant chemotherapy for early breast cancer

**DOI:** 10.1038/sj.bjc.6604171

**Published:** 2008-01-22

**Authors:** M Bhattacharyya, D Ryan, R Carpenter, S Vinnicombe, C J Gallagher

**Affiliations:** 1 Department of Medical Oncology, St Bartholomew's Hospital, West Smithfield, London EC1M 7BE, UK; 2 Department of Histopathology, St Bartholomew's Hospital, West Smithfield, London EC1M 7BE, UK; 3 Department of Surgery, St Bartholomew's Hospital, West Smithfield, London EC1M 7BE, UK; 4 Department of Radiology, St Bartholomew's Hospital, West Smithfield, London EC1M 7BE, UK

**Keywords:** breast cancer, MRI, neoadjuvant chemotherapy, breast-conserving surgery

## Abstract

Contrast-enhanced magnetic resonance imaging (MRI) was used to monitor the response of patients undergoing neoadjuvant chemotherapy for breast cancer with the aim of undergoing breast-conserving surgery (BCS). Patients were prospectively recruited to undergo MRI as well as conventional methods of clinical examination, mammography (MM) and ultrasonography (USS) and response was assessed by each of these methods. Thirty-two patients with primary breast cancer were recruited. Magnetic resonance imaging correlation with histopathological size (*r*=0.71) was superior to USS (*r*=0.65) and to MM where tumour size was not measurable following chemotherapy in 71% of patients. Magnetic resonance imaging had 87.5% sensitivity (95% CI=68–97%) and 50% specificity (95% CI=16–84%) for a PPV (positive predictive value) of 99.8% and NPV (negative predictive value) of 80% for the detection of residual invasive cancer. Magnetic resonance imaging displayed 80% sensitivity (95% CI=28.4–99.5%) and 89% specificity (95% CI=71–98%) to detect pathological pCR in the breast. Eighty-four per cent of recruited patients were identified as potentially suitable candidates for BCS following chemotherapy and of those choosing to accept BCS, breast conservation was achieved in 90.5%, or 65.6% of all patients. Of those who proceeded to BCS, 9.5% required a re-do mastectomy because of positive margins; however, no residual tumour was found on histological examination of mastectomy specimens. Magnetic resonance imaging appears to be superior to conventional methods for assessing pathological response and the low rate of re-operation for positive margins indicates a valuable role in aiding the decision to undergo BCS or mastectomy.

Pre-operative neoadjuvant chemotherapy has been an important development in the management of patients with early breast cancer. Although no more effective than post-operative treatment in improving survival, it has been shown to downstage large operable tumours, and increase the proportion of women who can be offered breast-conserving surgery (BCS), where previously a mastectomy would have been required (Hortobagyi *et al*, 1983). Neoadjuvant therapy also identifies the proportion of women achieving a complete pathological remission who are likely to have an excellent prognosis, and conversely, those for whom further treatment may be necessary (Feldman *et al*, 1986; Singletary *et al*, 2002).

Following neoadjuvant chemotherapy, accurate assessment of tumour size and location is necessary for planning the surgical management of the patient. There is a poor correlation between the histological appearances of the tumour and measurements obtained by physical examination, mammography (MM) or ultrasonography (USS). Radiological assessment is least accurate in younger women who most often desire BCS because their breast tissue is more dense making it more difficult to distinguish invasive cancer from residual *in situ* carcinoma and chemotherapy-induced fibrosis (Cocconi *et al*, 1984; Segel *et al*, 1988, Vinnicombe, 1996).

Limitations in tumour localisation may lead to a greater incidence of positive resection margins and the need for repeated excision or mastectomy in patients with a consequent failure to attain the desired goals of aesthetic breast conservation and optimum tumour control.

It has been suggested that breast magnetic resonance imaging (MRI) is more accurate in the diagnosis of primary breast cancer (Cocconi *et al*, 1984; Gilles *et al*, 1994; Abraham *et al*, 1996; Drew *et al*, 1999) and we therefore, wished to assess its use in the evaluation of tumour response and residual tumour size following chemotherapy when compared with conventional imaging. More accurate information may enable appropriate planning of surgery including BCS, minimising re-excision rates while maintaining the efficacy and possibility of cure.

## 

### Aims

This study was designed to assess the usefulness of MRI in measuring response to neoadjuvant chemotherapy and maximising the successful use of BCS. Magnetic resonance imaging was compared with MM and USS to assess its ability to predict residual tumour size and viability. Breast surgery outcomes were recorded and the histopathological report was used as the gold standard.

## MATERIALS AND METHODS

### Inclusion criteria

Patients with histologically confirmed invasive breast cancer by core needle biopsy were prospectively recruited to the study if they had operable breast cancer and wished to have BCS but were judged likely to benefit from pre-operative chemotherapy to downstage the tumour. Neoadjuvant chemotherapy was offered if there were features thought to predict for a higher risk of recurrence, following breast conservation, or likely failure to achieve aesthetic breast conservation; these features included the following: (a) tumours 4 cm or greater in maximum diameter, (b) relatively large tumours in women those have small breasts (c) clinical nodal involvement confirmed by fine-needle aspiration.

### Exclusion criteria

Patients with locally advanced breast carcinoma and inflammatory breast cancers were excluded. All patients were assessed by chest X-ray, bone scan and liver USS and were excluded if these were positive for metastatic spread.

### Treatment

Patients received anthracycline-based neoadjuvant chemotherapy for six cycles at 21-day intervals with doxorubicin (60 mg m^−2^) or epirubicin (90 mg m^−2^) and cyclophosphamide (600 mg m^−2^).

### Assessment

All patients had a regular clinical examination with each cycle of treatment, and maximum tumour diameters in two perpendicular directions were measured clinically at each visit. Patients who failed to respond after two cycles, or, who progressed clinically while receiving chemotherapy and received alternative chemotherapy and salvage mastectomy, were excluded from the imaging analysis, as imaging was incomplete. Triple breast imaging, with MM, USS and MRI, was performed within the 4 weeks before commencing chemotherapy and at 4 weeks after the sixth course of chemotherapy was completed.

Mammograms were obtained on standard mammographic units (full field digital Lorad Selenia). Mediolateral, oblique and craniocaudal views were obtained with magnification views of any microcalcification as necessary. The size of any mass was measured in two perpendicular directions and an assessment made of overall shape and border characteristic of the mass.

Examinations assessed by USS were carried out pre- and post-treatment with high-frequency linear phased probes, centre frequency 12 mHz, (Acuson Sequoia) and again maximum diameters of any masses were recorded. Volumes were not calculated routinely. Care was taken to ensure that measurements were in similar planes and orientations at both ultrasound and MM.

Breast MRI was carried out on a 1.5 T GE Signa magnet using a dedicated double breast coil. Axial and sagittal T1-weighted spin echo sequences were followed by a semi-dynamic 3D T1-weighted gradient echo sequence pre- and post-intravenous gadolinium-DTPA (16 mmol kg^−1^). Morphology (shape, border characteristics) and size of any mass was recorded by one observer, blinded to the results of MM and USS. Time–intensity curves were routinely plotted and characterised. The MR slice thickness, on the dynamic and high-resolution post-contrast scans, was 2–3 mm. The interval between conventional imaging and breast MRI was under 2 weeks.

On the basis of the physical and radiological examination after chemotherapy, and patient choice, the patients were advised by the multi-disciplinary team to proceed to simple mastectomy or breast-conserving segmental mastectomy and axillary dissection. Pathological size and the composition of any residual mass at outcome surgery was compared with the maximum tumour diameters observed with imaging, using the Pearson's correlation coefficient.

## RESULTS

### Baseline characteristics

A total of 32 patients aged between 24 and 60 (median age 42 years) satisfied all inclusion criteria for the imaging study. Twenty-two (69%) were pre-menopausal and 10 (31%) were peri- or post-menopausal. Patients presented with Stage 1 (6%), Stage 2 (62%) and Stage 3 (31%) disease and median tumour size at presentation was 4.75 cm (range=2–8 cm). Tumours were staged using the TNM classification (Singletary *et al*, 2002). All completed six cycles of anthracycline-based chemotherapy.

### Response to chemotherapy

All 32 patients had MRI scans preceding neoadjuvant chemotherapy and at the completion of chemotherapy. Twenty-eight patients had mammograms and 30 had ultrasound scans pre- and post-chemotherapy. Pre- and post-chemotherapy measurements, for each imaging modality, are shown for each patient (Table 1). Response was assessed radiologically by RECIST criteria (Therasse *et al*, 2000) and clinical response, based on bi-dimensional measurement (Table 2). Mammography data were incomplete as it was not possible to measure the size of tumour after chemotherapy in 18 patients (56%) because the tumour margins were no longer assessable.

### Correlation with histology

The pathological complete response rate (pCR) in the breast in this study was 12.5%. Seven patients had a complete radiological response as assessed by MRI. Pathological complete response was present in four of these patients and residual foci of invasive disease seen in the remaining three patients measuring from 0.1 to 0.6 cm in diameter. Histological size and the size assessed by USS in these patients is shown (Table 3). It was therefore possible to detect pCR using MRI; however, MRI overestimated the rate of pCR.

The performance of the MRI scan in detecting residual invasive cancer was sensitivity 87.5% (95% CI=67.6–97.3%), specificity 50% (95% CI=15.7–84.2%) for a positive predictive value (PPV) of 99.8% and a negative predictive value (NPV) of 80%. For the detection of pCR the MRI scan performance was sensitivity 80% (95% CI=28.4–99.5%), specificity 89% (95% CI=70.8–97.6%) for a PPV of 56% and an NPV of 96%.

In four patients, the post-chemotherapy MRI residual tumour size was over 1 cm larger than pathological size. In each of these cases, histological evaluation revealed the presence of DCIS, which had an enhancement pattern on MRI that could not be differentiated from the invasive component (Table 3).

Overall, MRI correlation with histopathological size (*r*=0.71) was superior to USS correlation (*r*=0.65). Correlation could not be obtained for MM, as tumour size was not measurable in more than 50% of patients following chemotherapy.

### Surgical outcome

Following neoadjuvant chemotherapy, 27 (84%) patients were judged potentially suitable for BCS, on the basis of clinical examination and radiological assessment. Of these, 21 proceeded to breast conservation and 4 patients changed their mind and chose to have mastectomy. Two patients who had no evidence of residual tumour on physical examination or radiological assessment underwent localisation biopsy and were therefore offered radiotherapy alone; however, localisation biopsy confirmed the absence of tumour.

Of the 21 patients who had BCS, 2 patients (10%) required subsequent mastectomy because of positive excision margins following the initial surgery. In one of these patients, the post-chemotherapy pre-operative MRI showed a partial response with a 3.6 by 2.7 cm area of gradual enhancement postero-lateral to the nipple thought likely to represent residual invasive disease whereas MM was normal and the USS showed a 0.3 cm hypoechoic lesion. Histology of the initial segmental mastectomy showed a 0.1-cm invasive tumour involving the margin and the patient proceeded to mastectomy, the histology of which showed no residual tumour. In the second patient, the MRI scan reported a partial response with an enhancing focus measuring 1.0 by 0.7 cm with no discernible tumour mass; the surrounding parenchyma displayed non-malignant enhancement, possibly due to chemotherapy-induced fibrosis or a residual intraduct component. In this patient, USS had shown a 1.7-cm mass. Histology of the segmental mastectomy showed a multifocal grade 2 infiltrating ductal carcinoma measuring 1.8 cm with associated low-grade cribriform DCIS with a tumour deposit in the medial en face surface. Histology from the mastectomy however showed no residual invasive cancer.

Nine patients had a mastectomy within this study. The reasons for proceeding to mastectomy are given and include patient's choice, position of residual tumour and identification of residual multifocal tumour (Table 4).

In the three patients, who chose to have mastectomy, one had a complete response on MRI and histology and one had a small 1.5 cm tumour as predicted on MRI. However, in one patient, the post-chemotherapy tumour size on MRI was 5.6 cm preoperatively whereas the histology showed a 1.8-cm Grade 2 ductal carcinoma. In this patient, the MRI scan had not distinguished invasive tumour from the extensive DCIS seen on the histopathological specimen.

It was not possible to calculate the sensitivity and specificity of the MRI in predicting successful BCS as the MRI result was used to guide the decision; thus, no patients were considered for BCS in whom MRI was not thought to be favourable. This was further confounded by two patients who did not have surgery, and three patients who despite a favourable MRI result chose to have mastectomy.

## DISCUSSION

The use of breast MRI scanning in this group of women being considered for BCS after neoadjuvant chemotherapy led to a more accurate preoperative assessment of the response than MM or ultrasound. Magnetic resonance imaging detection of residual invasive disease in comparison with operative histology had a PPV of 99.8% and an NPV of 80%. However, as might be expected MRI did not perform as well in detecting complete pathological remissions with a PPV of 56% though the NPV was 96%.

The MRI correlation with histopathological size (*r*=0.71) is similar to that found previously with correlations of *r*=0.75 (Rosen *et al*, 2003) and 0.72 (Martincich *et al*, 2004) reported. Other groups have reported correlations of *r*=0.6 (Partridge *et al*, 2002) and *r*=0.982 (Cheung *et al*, 2003).

Magnetic resonance imaging was superior to ultrasound for assessment of tumour size (*r*=0.61) and to physical examination. Mammography was found to be unreliable in the assessment of preoperative tumour size because of the inability to define the tumour margins.

All patients were fully informed about their treatment options and were supported by a breast care nurse so that they could participate in the decisions about their treatment. As a result of our policy of neoadjuvant chemotherapy (that excluded all T4 tumours) BCS was achieved in 19 out of 32 (59.4%) patients for whom mastectomy would have otherwise been necessary. Mastectomy was still required in eight (25%) patients to achieve optimum local tumour control. The remaining five patients (15.6%) were rendered suitable for BCS but chose mastectomy (three patients),or radiotherapy only (two patients).

Breast MRI has been found to be very sensitive for the detection of primary invasive breast cancer but less is known about the accuracy of MRI for detecting residual disease after chemotherapy as cytotoxic agents may affect the dynamics of contrast uptake (Gilles *et al*, 1994; Orel *et al*, 1995; Hunt *et al*, 1996; Fisher *et al*, 1998; Partridge *et al*, 2002; Bedrosian *et al*, 2003).

In breast conservation, positive margins are associated with an increased long-term risk of cancer recurrence in the ipsilateral breast (Fourquet *et al*, 1989; Anscher *et al*, 1993; Pittinger *et al*, 1994; Dewar *et al*, 1995; Gage *et al*, 1996; Wazer *et al*, 1998; Freedman *et al*, 1999; Cowen *et al*, 2000) and, therefore, patients with positive margins, following BCS, require further surgery. In our series, the re-operation rate was 9.5% (two patients) but no histological evidence of residual tumour was found on the mastectomy specimen.

Accurate assessment of tumour response to chemotherapy may correctly identify which patients are suitable candidates for BCS, minimising the rates of re-excision surgery, thereby minimising risk and distress to the patient caused by repeated surgical procedures. Physical examination and conventional imaging are unable to reliably predict the presence or extent of residual or recurrent disease.

Several studies have suggested that MRI is a more accurate method of delineating residual tumour following neoadjuvant chemotherapy than clinical, ultrasound or mammographic assessment (Boetes *et al*, 1995; Abraham *et al*, 1996; Drew *et al*, 1999, 2001; Fischer *et al*, 1999; Balu-Maestro *et al*, 2002; Rieber *et al*, 2002; Cheung *et al*, 2003; Rosen *et al*, 2003; Martincich *et al*, 2004; Warren *et al*, 2004). Chemotherapy-induced fibrosis has been shown to impair the evaluation of tumour by conventional radiological methods and physical examination (Cocconi *et al*, 1984; Segel *et al*, 1988). We also found that similar factors together with the presence of extensive DCIS limited to the interpretation of the MRI scans.

The final decision to undertake BCS is a complex mixture of surgical judgement and patient's expectations and desires, so we were not able to assess in this study how much it had been advanced by MRI scanning. The low rate of re operation for positive margins (2 out of 21 9.52%) may be one measure of success and compares favourably with other reported series however longer term follow-up will be necessary to assess local recurrence rates and overall survival.

## Figures and Tables

**Table 1:** Clinical features: sizes of tumours pre-chemotherapy, post-chemotherapy and after surgery

			**Max diameter pre-chemo (cm)**				**Max diameter post-chemo (cm)**				
	**Age (years)**	**Stage**	**Clinical**	**MRI**	**Mammo**	**USS**	**MRI**	**Mammo**	**USS**	**Surgery**	**Histology (cm)**
1	24	T2N0	5	4.5	NA	4.5	3	Not measurable	2.5	BCS	2.5
2	41	T3N0	6	3	3.5	2.8	2.5	3.5	2	BCS	2.3
3	52	T2N0	4	3.5	3	3.1	3.4	Not measurable	2.5	BCS	4
4	48	T2N0	4	3.5	NA	3	0	Not measurable	2	BCS	0.6
5	41	T3N1	5	4.5	2	4	1.8	1.5	1.7	BCS then re-do	1.8
6	55	T3N1	6	5	5	NA	5.6	Not measurable	3.9	Mastectomy	1.8
7	42	T2N0	5	4	5	NA	0	0	0.3	BCS then re-do	0.1
8	43	T2N0	4	2.3	5	5	2	Not measurable	1.5	BCS	1.5
9	42	T2N1	5	2.7	NA	2.2	3	Not measurable	2.2	BCS	1.4
10	40	T2N0	3	2	NA	2.3	1.3	1.8	0.8	BCS	1.3
11	49	T1N1	2	2	NA	0.6	0	0.6	0.5	Mastectomy	0
12	56	T2N2	3	2.3	2	2	1.3	Not measurable	1	BCS	0.95
13	35	T2N1	4.5	6	2	4	1.4	Not measurable	0.8	Mastectomy	4
14	49	T2N1	3	3	3	2.8	2	Not measurable	2.5	BCS	0 (DCIS)
15	55	T3N1	6	2	6	3	0	0	0	BCS	0
16	47	T2N1	5	2.3	NA	NA	0	0	0.3	Radiotherapy only	0
17	60	T2N1	3	2.5	2.5	2.5	0	1	0	Radiotherapy only	0
18	36	T2N0	4	2.4	0	1.2	2.5	Not measurable	2.8	BCS	2.4
19	50	T2NX	2	2.2	1	2.1	0	0	0	BCS	0.6
20	40	T2N1	2	3	2	2.5	1.5	Not measurable	1.3	Mastectomy	1.1
21	35	T3N1	6	4.5	3	4	1.5	Not measurable	1.2	BCS	1.4
22	55	T3N1	5	5	NA	4	3	4	3.3	Mastectomy	6.7
23	33	T2N0	3.5	4	NA	3.7	1	0	0	BCS	0.1
24	37	T3N0	8	3.5	4	3	2	Not measurable	1.9	BCS	2.7
25	35	T1N0	2	2.8	2	2	1.2	Not measurable	NA	Mastectomy	1.5
26	47	T3N1	4	5	4	3.9	2.5	Not measurable	1.3	BCS	1.5
27	53	T3N1	6	4	3	2	1.3	0	0	Mastectomy	0.1
28	33	T2N0	2	3.5	NA	3	0.9	0	0.7	BCS	1.2
29	33	T3N1	6	4	5.5	5.2	2.5	Not measurable	2.8	BCS	4
30	25	T3N0	5	4	5.5	5	4	2	2.6	BCS	3.5
31	31	T3N1	5	4	NA	4.5	1	Not measurable	1.1	Mastectomy	1.9
32	45	T2N0	5	3	NA	3.7	2.5	Not measurable	0.4	Mastectomy	2.8

**Table 2:** Response to chemotherapy as measured clinically, by MRI, mammography and ultrasound, as assessed by RECIST criteria

**Response**	**Clinical**	**MRI**	**MM**	**USS**
Complete response (CR)	4 (12.5%)	7 (22%)	5 (16%)	5 (16%)
Partial response (PR)	26 (81%)	17 (53%)	1 (3%)	17 (53%)
Stable disease (SD)	2 (6%)	8 (25%)	2 (6%)	4 (12.5%)
Progressive disease (PD)	0	0	0	0
Not measurable	0	0	20 (62.5%)	4 (12.5%)

*n*=32 patients.

**Table 3:** Cases where MRI residual abnormality was greater than histological tumour: details of MRI and histology reports

**Patient**	**MRI size (cm)**	**MRI report**	**Histology invasive cancer size (cm)**	**Histology report**
6	5.6	Reduced tumour volume noted	1.8	Grade 2 infiltrating ductal carcinoma with surrounding high grade DCIS
9	3	Main tumour mass 3 cm, satellite areas and intraduct component	1.4	Grade 2 ductal carcinoma
14	2	2 cm mass, striking surrounding distortion possible desmoplastic reaction or intraduct component.	0	Residual DCIS only
26	2.5	Not all of mass enhances. Residual enhancing focus 10 mm. Enhancement of surrounding parenchyma either chemotherapy-induced fibrosis or residual intraductal component	1.5	Grade 3 infiltrating ductal carcinoma; surrounding high grade DCIS

**Table 4:** MRI responses in patients who proceeded to mastectomy and reasons for mastectomy

**Patient**	**MRI response**	**MRI assessment**	**Reason for mastectomy**
6	SD	Ineligible	Large residual tumour
11	CR	Eligible	Patient choice
13	PR	Ineligible	Residual tumour too close to nipple
20	PR	Ineligible	Residual tumour too close to nipple
22	PR	Eligible	Patient delayed surgery – clinical progression
25	PR	Eligible	Patient choice
27	PR	Eligible	Possible skin tethering clinically noted at finish of treatment
31	CR	Ineligible	Multifocal at finish of chemotherapy
32	PR	Ineligible	Residual multifocal tumour
5	PR	Eligible	Positive excision margins at BCS
7	PR	Eligible	Positive excision margins at BCS
